# The Comparison of Laparoscopic Colorectal Resection with Natural Orifice Specimen Extraction versus Mini-Laparotomy Specimen Extraction for Colorectal Tumours: A Systematic Review and Meta-Analysis of Short-Term Outcomes

**DOI:** 10.1155/2020/6204264

**Published:** 2020-01-31

**Authors:** Jun He, Jun-Feng Hu, Shu-Xian Shao, Hai-Bo Yao, Xiu-Feng Zhang, Guan-Gen Yang, Zhong Shen

**Affiliations:** ^1^Department of Colorectal Surgery, Hangzhou Third Hospital, Hangzhou 310009, Zhejiang, China; ^2^Department of Gastroenterology and Pancreatic Surgery, Zhejiang Provincial People's Hospital, Hangzhou 310014, Zhejiang, China

## Abstract

**Aim:**

The aims of this study were to compare the short-term outcomes of natural orifice specimen extraction surgery (NOSES) and conventional laparoscopic surgery (CLAPS) for colorectal tumours and to evaluate the safety and feasibility of NOSES in colorectal resection.

**Methods:**

A literature review was performed on the PubMed, Cochrane Library, and Embase databases up to March 2019. Papers conforming to the inclusion criteria were used for further analysis. The short-term outcomes included intraoperative outcomes and postoperative recovery results. The weighted mean difference (WMD) was calculated for continuous outcomes and odds ratio (OR) for dichotomous results. Study quality was evaluated using the Newcastle-Ottawa Quality Assessment Scale (NOS) or the 6-item Jadad scale.

**Results:**

Eight studies comprising 686 patients met the inclusion criteria. Compared with CLAPS, NOSES had more advantages in terms of postoperative complications, postoperative pain, recovery of gastrointestinal function, duration of hospital stay, and cosmetic results. The lymph nodes harvested and intraoperative blood loss in NOSES were comparable with CLAPS; however, a prolonged operative time was observed in NOSES.

**Conclusions:**

NOSES was shown to be a safe and viable alternative to CLAPS in colorectal oncology in terms of short-term results. Further long-term and randomized trials are required.

## 1. Introduction

Since it emerged in the 1990s, laparoscopic proctocolectomy has been widely used for the treatment of various colorectal diseases including tumours [1–4]. Compared with open surgery, laparoscopic surgery achieves the same oncological outcomes and accelerates postoperative recovery [5, 6]. However, conventional laparoscopic surgery (CLAPS) still needs a mini-laparotomy for specimen harvest. Because of the additional 5-6 cm incision, incision-related complications such as postoperative pain, incision infection, abdominal wall scar, and even incision hernia are problematic [7–10]. To mitigate these complications, a novel, minimally invasive surgery known as natural orifice specimen extraction surgery (NOSES) has been increasingly used worldwide [11]. Studies have reported successful results in terms of laparoscopy with natural orifice specimen extraction, as with this technique, an auxiliary incision has been eliminated [12–14]. Compared with CLAPS, the main features of NOSES in colorectal surgery are complete intraperitoneal anastomosis and specimen extraction from natural orifice [14–17]. This innovative technique is regarded as a step towards minimally invasive surgery.

A number of studies have assessed NOSES and CLAPS in terms of their short-term results, safety, and efficacy, but there remains uncertainty [11]. Notably, a recent review comparing NOSES with CLAPS has been published [18]. However, it recruited many nontumour studies without radical resection and did not report pathological results. Moreover, several comparative studies have been published in recent years. In the present study, we undertook a systematic review and meta-analysis to compare the short-term results between NOSES and CLAPS and to provide convincing evidence for clinical practice in colorectal oncology.

## 2. Methods

### 2.1. Search Strategy

A systematic search of the articles comparing of NOSES and CLAPS for colorectal tumours was performed on the PubMed (MEDLINE), Cochrane Library (CENTRAL) and Embase databases up to March 2019. The following main terms were used: “Colorectal Neoplasms,” “Rectal Neoplasms,” “Colonic Neoplasms,” “Laparoscopy,” “natural orifice specimen extraction,” “transvaginal specimen extraction,” “transanal specimen extraction,” “transrectal specimen extraction,” “transcolonic specimen extraction,” and “natural orifice transluminal endoscopic surgery.” The search strategy of Embase was presented in S1 Text. There were some distinctions in the specific search strategy used between different databases. Potentially relevant studies were also found by screening the references of related literature.

### 2.2. Inclusion and Exclusion Criteria

Studies had to meet the following criteria: (1) NOSES was compared with CLAPS for colorectal tumour; (2) radical resection was performed with lymph node results; (3) at least 2 outcomes of interest were reported and characteristic baselines were comparable; (4) the most comprehensive research was recruited when overlapping researches were found by the same team; and (5) full text was available in English. Studies were excluded if they met one of the following criteria: review, conference abstract, non-English, full-text unavailable, nontumour disease, characteristic baseline imbalance, robotic surgery, or single-port laparoscopic surgery.

### 2.3. Data Extraction and Quality Assessment

All studies were assessed by two independent reviewers (Jun He and Jun-Feng Hu). The data extracted for analysis comprised three parts: patient characteristics (age, sex, body mass index (BMI), and American Society of Anesthesiologists (ASA) score), intraoperative outcomes (operation time, estimated blood loss, and lymph nodes harvested), and postoperative recovery (pain score, additional analgesics, gastrointestinal function, complications, duration of hospital stay, and cosmetic results). The quality of the included retrospective comparative studies was assessed by utilizing the Newcastle-Ottawa Quality Assessment Scale (NOS) [19], and studies achieving six or more stars were deemed high quality. Randomized controlled trials were evaluated by using the 6-item Jadad scale [20], and studies with a score of 5 or more were eligible. Discussions were held to eliminate discrepancies by the two reviewers.

### 2.4. Statistical Analysis

STATA 12.0 (Stata Corp) was used for data analysis. The weighted mean difference (WMD) was applied to evaluate continuous variables, and the odds ratio (OR) was utilized to calculate dichotomous variables. The estimated values were calculated by using formulas designed by Hozo et al. when the mean and standard deviation (SD) were not provided [21]. A random-effects model based on DerSimonian and Laird's method was adopted because of the clinical heterogeneity of observational studies. The Mantel–Haenszel statistical method was applied to dichotomous outcomes, and inverse variance was applied to continuous outcomes. Heterogeneity was measured using the *χ*^2^ test and *I*^2^ index. In addition, heterogeneity was considered significant when *I*^2^ > 50% (*P* < 0.1), and a sensitivity analysis and a subgroup analysis were conducted to assess the source of heterogeneity. Publication bias was examined with a funnel plot. *P* < 0.05 was considered statistically significant.

## 3. Results

### 3.1. Description of Included Studies and Patient Characteristics

**Figure 1:**
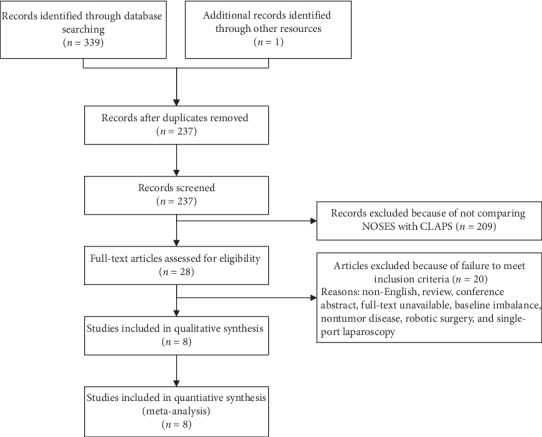
Flowchart of studies included in the meta-analysis.

The preliminary literature retrievals identified 339 studies. Eight studies met the inclusion criteria for further meta-analysis [10, 13, 22–27] (Figure 1). Among these, seven were retrospective studies and one was a prospective randomized controlled trial. The characteristics of the included studies are shown in Table 1. A total of 686 patients were recruited in those studies. There were 293 patients in the NOSES group and 393 patients in the CLAPS group. The characteristics of the patients are presented in Table 2.

### 3.2. Surgical and Pathological Outcomes

#### 3.2.1. Operation Time and Intraoperative Blood Loss

**Figure 2:**
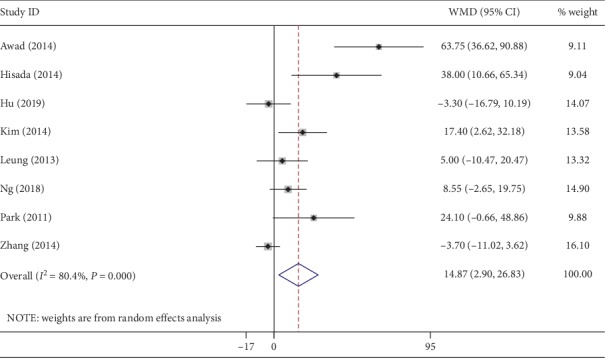
Forest plot comparing operation time in the NOSES group and the CLAPS group.

**Figure 3:**
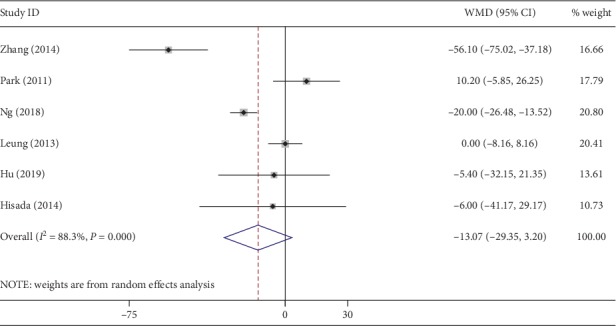
Forest plot comparing intraoperative blood loss in the NOSES group and the CLAPS group.

All included studies reported the operation time. The meta-analysis revealed that the operation time of the NOSES group was significantly longer than that of the CLAPS group (WMD, 14.87 min; 95% CI, 2.90∼26.83 min; *P*=0.01) (Figure 2). However, an obvious heterogeneity (*I*^2^ = 80%) existed in the operation time. Of the 8 studies, 6 reported intraoperative estimated blood loss. The blood loss was not significantly different between the two groups (WMD, −13.07 ml; 95% CI, −29.35∼3.20 ml; *P*=0.12) (Figure 3). Similarly, a high heterogeneity (*I*^2^ = 88%) was observed among the included studies.

#### 3.2.2. Lymph Nodes Harvested

**Figure 4:**
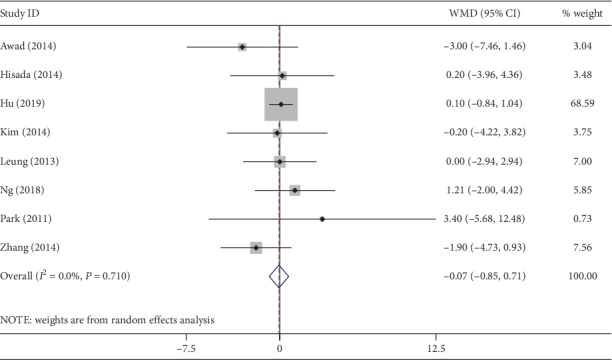
Forest plot comparing lymph nodes harvested in the NOSES group and the CLAPS group.

All studies provided data about the number of totally dissected lymph nodes. No significant difference was observed between the two groups with respect to lymph nodes harvested (WMD, −0.07; 95% CI, −0.85∼0.71; *P*=0.86) (Figure 4). No heterogeneity was detected (*I*^2^ = 0%).

### 3.3. Postoperative Recovery

#### 3.3.1. Postoperative Complications

**Figure 5:**
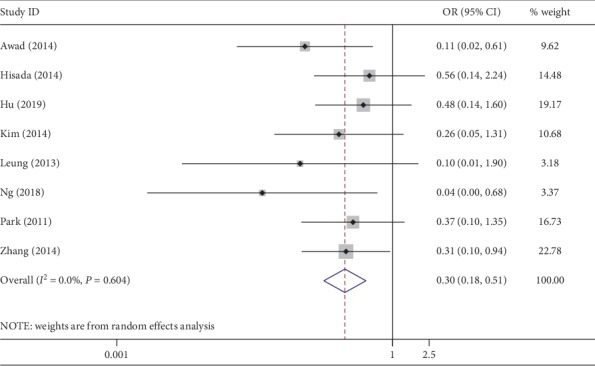
Forest plot comparing postoperative complications in the NOSES group and the CLAPS group.

All the recruited studies reported the incidence of short-term complications after surgery. This meta-analysis showed that patients in the NOSES group had a statistically lower rate of complications than those in the CLAPS group (OR, 0.3; 95% CI, 0.18∼0.51; *P* < 0.01) (Figure 5). The result was reasonably precise and statistically homogeneous (*I*^2^ = 0%).

#### 3.3.2. Postoperative Pain and Additional Analgesics

**Figure 6:**
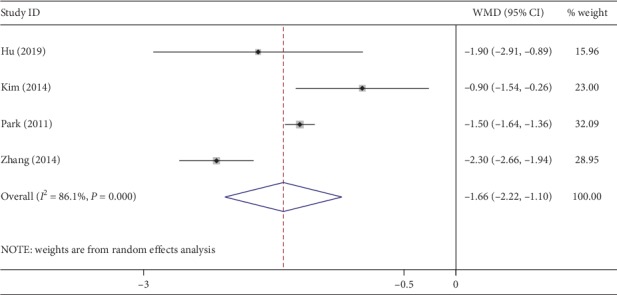
Forest plot comparing postoperative pain scores in the NOSES group and the CLAPS group.

**Figure 7:**
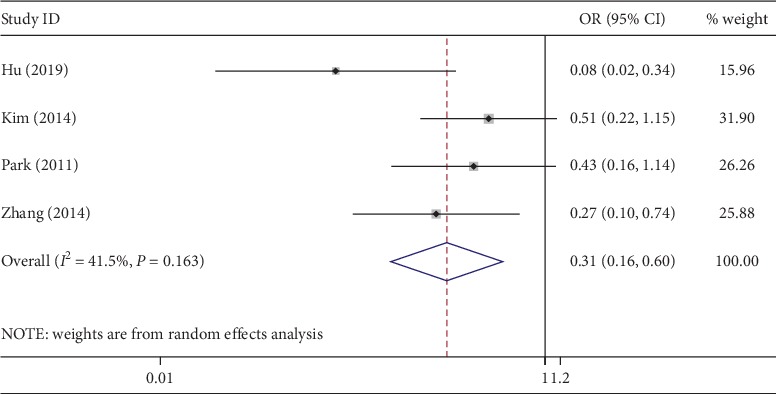
Forest plot comparing usage rate of additional analgesics in the NOSES group and the CLAPS group.

Of the 8 studies, 7 evaluated postoperative pain using a visual analogue scale (VAS) [28]. However, only 4 studies recorded pain scores at 24 h after surgery. NOSES achieved significantly lower pain scores (WMD, −1.66; 95% CI, −2.22∼−1.10; *P* < 0.01) (Figure 6), though with a high heterogeneity (*I*^2^ = 86%). According to the above four studies, the rate of use of additional analgesics in CLAPS was much higher (OR, 0.31; 95% CI, 0.16∼0.60; *P* < 0.01) (Figure 7), with low heterogeneity (*I*^2^ = 41%).

#### 3.3.3. Recovery of Gastrointestinal Function

**Figure 8:**
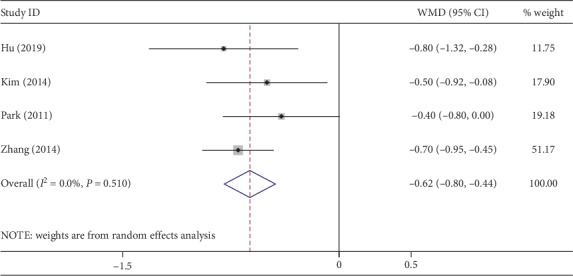
Forest plot comparing the time to first passage of flatus in the NOSES group and the CLAPS group.

**Figure 9:**
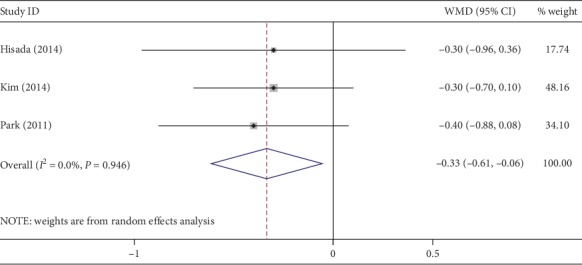
Forest plot comparing the first time to oral ingestion in the NOSES group and the CLAPS group.

Four studies provided data about gas passage after surgery. The time to first passage of flatus was earlier in NOSES (WMD, −0.62; 95% CI, −0.80∼−0.44; *P* < 0.01) (Figure 8). No heterogeneity was observed (*I*^2^ = 0%). Three studies described the first time to oral ingestion. The time to regular diet was shorter with NOSES (WMD, −0.33; 95% CI, −0.61∼−0.06; *P*=0.02) (Figure 9). No heterogeneity was detected (*I*^2^ = 0%).

#### 3.3.4. Length of Hospital Stay

**Figure 10:**
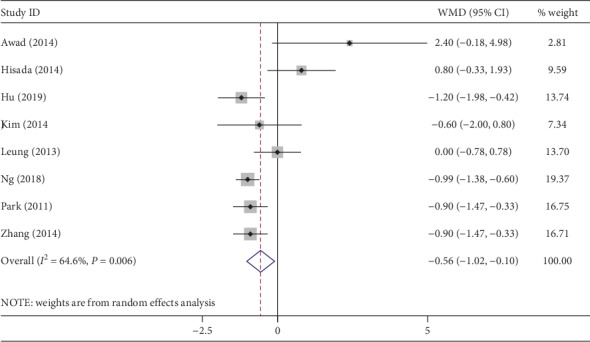
Forest plot comparing duration of hospital stay in the NOSES group and the CLAPS group.

The duration of hospital stay was recorded in all studies. Compared with CLAPS, the hospital stay with NOSES was shorter (WMD, −0.56 days; 95% CI, −1.02∼−0.10 days; *P*=0.02) (Figure 10). However, high heterogeneity was noted (*I*^2^ = 65%).

#### 3.3.5. Aesthetics

**Figure 11:**
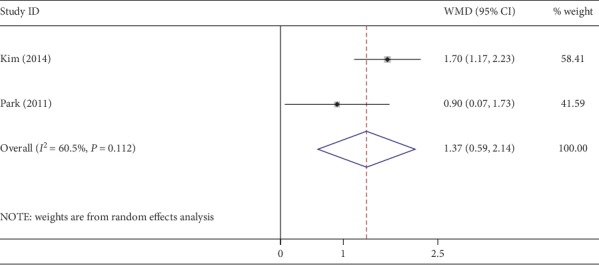
Forest plot comparing cosmetic scores in the NOSES group and the CLAPS group.

Four studies reported the cosmetic results, and only 2 studies offered cosmetic scores (0 to 10, 0 as poor satisfaction and 10 as excellent satisfaction) and standard deviations. The pooled data showed that the NOSES group gained better aesthetic properties (WMD, 1.37; 95% CI, 0.59∼2.14; *P* < 0.01) (Figure 11). High heterogeneity was detected (*I*^2^ = 60%).

### 3.4. Sensitivity and Subgroup Analysis

Sources of heterogeneity were investigated by conducting sensitivity and subgroup analyses. The sensitivity analysis was performed by the sequential removal of individual articles. In addition, subgroup meta-analysis based on the following items was also performed: (1) specimen extraction route (SER), (2) sample size (NOSES group sample size <35), and (3) publication year. The sources of heterogeneity in operative time, intraoperative blood loss, hospital stay, and pain scores were not found by sensitivity analysis. Meanwhile, subgroup analyses demonstrated that SER, sample size, and publication year were all not associated with the heterogeneity. The results of subgroup analysis are shown in S1–S4 figures.

### 3.5. Publication Bias

**Figure 12:**
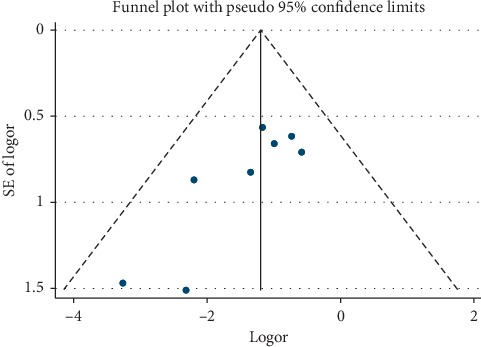
Funnel plot of the postoperative complications.

A funnel plot analysis of postoperative complications was performed to detect publication bias. It showed that all the inclusive studies were within the 95% confidence interval, and no publication bias was found (Figure 12).

## 4. Discussion

Over the past two decades, laparoscopic techniques have been increasingly applied for colorectal resection, and the safety and efficacy of CLAPS have been well demonstrated [5, 6, 29]. In pursuit of optimized outcomes, NOSES without mini-laparotomy incision has been introduced to reduce postoperative morbidity and to improve recovery. As laparoscopic techniques and devices have progressed, NOSES has been well accepted by colorectal surgeons and patients in recent years [11, 17, 30]. This meta-analysis demonstrated the safety and feasibility of NOSES in the treatment of colorectal tumours. The pooled results revealed that complete laparoscopic colorectal resection with NOSES has more advantages in terms of postoperative recovery, postoperative pain, aesthetics, and complications; however, it was associated with longer operative time.

Previous studies have already revealed that the operative time of NOSES is prolonged [10, 13, 27]. The results of this meta-analysis confirmed that patients in group NOSES experienced a significantly longer operative time than those in group CLAPS. There are some possible causes for this increased time. First, the procedure of total intraperitoneal anastomosis may be prolonged [10]. Second, the learning curve and surgeons' familiarity with laparoscopic techniques are also important reasons for longer surgical duration. After several practice procedures, a steady reduction in the operative time for NOSES was noticed [13, 23]. Intraoperative bleeding volume and conversion rate are two indicators to evaluate the safety of laparoscopic surgery. The analysis showed that there was no statistically significant difference between the two groups in intraoperative haemorrhage. Seven included studies recorded the conversion rate of NOSES, and no conversion to open surgery was reported. However, a mini-laparotomy was used for specimen extraction in three patients because of the bulky specimen [12, 27]. Hence, the tumour size in NOSES should be strictly restricted.

The number of lymph nodes dissected is closely related to overall survival in cancer patients after surgery [31]. The results of this meta-analysis revealed that the groups were comparable in terms of lymph node retrieval. According to studies with follow-up, local recurrence was not observed during the follow-up period [10, 24].

Postoperative complications are among the crucial indicators of the safety of emerging techniques. Our meta-analysis revealed that the postoperative morbidity in the NOSES group was significantly lower than that in the CLAPS group. This could be attributed in great part to the reduction of incision-related complications such as incision infection. The utilization of natural orifice points to eliminate a large incision in NOSES is the primary cause. In addition, studies revealed that the risk of peritoneal bacterial contamination and neoplasm seeding in NOSES were comparable with that in CLAPS [10, 32–34]. In routine clinical practice, some measures can be recommended against contaminated or seeding-related complications, including bowel preparation, prophylactic antibiotic use, peritoneal lavage, and the use of sterile protection devices when retrieving specimen [30]. On account of anus and vagina as extraction routes, postoperative anal incontinence and dyspareunia have become a topic of focus. Based on previous studies, the incidence of these two complications is low, and the symptoms are usually mild and reversible [10, 26].

Minimally invasive surgery has been shown to have superior results, especially in postoperative recovery [35]. In this meta-analysis, patients in the NOSES group experienced faster recovery of gastrointestinal function (early first passage of flatus and regular diet) than those in the CLAPS group. In addition, significantly decreased postoperative pain was observed in the NOSES group. This could be attributed to the trauma in NOSES being further reduced [36]. Owing to less pain, the need for additional analgesics was also reduced. Al-Ghazal et al. observed that the postoperative cosmetic result acquired had a remarkable bearing on psychosocial morbidity [37]. The aesthetic advantages of NOSES over the conventional laparoscopic techniques were notable. Because of the scarless healing, patients in the NOSES group experienced higher satisfaction. Whether the aesthetics in colorectal surgery influenced the psychological outcome and prognosis is worth studying further. With respect to the duration of hospitalization, these pooled data revealed that the length of hospital stay in the NOSES group was shorter than that in the CLAPS group, potentially due to the benefits of fewer postoperative complications and accelerated recovery in NOSES.

Our research has several limitations. First, the quality of the meta-analysis is most often determined by original studies. In this pooled study, observational comparative studies accounted for most studies, and only one eligible randomized controlled trial was included. Large-sample and high-quality trials are required to strengthen our results. Second, heterogeneity was observed in some results. Therefore, the random-effect model was adopted. Additionally, sensitivity and subgroup analyses were conducted to explore the possible sources of heterogeneity. Despite the heterogeneity, most of the results of this pooled analysis were stable and conclusive. Third, some results recruited limited studies, which may reduce the persuasion of the results. Hence, more comprehensive studies containing adequate intraoperative outcomes, postoperative parameters, pathological results, and long-term outcomes are needed. Besides, the NOSES technique also has some inherent limitations and the indication of NOSES should follow the indication of conventional laparoscopic colorectal resection. The application of this technique is restricted by surgeon, patients' gender, and tumour size. The NOSES should be operated by experienced surgeons with conventional laparoscopic colorectal surgery. Transanal NOSES suits for male or female patients, and the tumour diameter is recommended less than 3 cm. However, transvaginal NOSES is only applied for female patients, and the tumour diameter is limited within 5 cm. [30] Although the safety and feasibility are well demonstrated, these constraints must be taken into consideration before the implementation of NOSES in colorectal surgery.

## 5. Conclusions

In summary, the short-term perioperative results of NOSES were confirmed to be comparable with those of CLAPS. NOSES was superior to CLAPS for radical colorectal resection in terms of overall postoperative complications, recovery of gastrointestinal function, postoperative pain, aesthetics, and hospital stay. In addition, a prolonged operative time was also observed in NOSES. Considering these insufficiencies of the study, further multicenter, large-sample, prospective randomized controlled, and long-term follow-up studies are needed. To sum up, the safety and feasibility of NOSES for colorectal tumours was demonstrated.

## Figures and Tables

**Table 1:** Characteristics of the included studies.

Author	Year	Region	Study design	Patients (*n*)		NOS score	Specimen extraction site
				NOSES	CLAPS		
Awad et al.	2014	USA	RCNS	20	20	8	Vaginal
Hisada et al.	2014	Japan	RCNS	20	50	6	Anal
Hu et al.	2019	China	RCNS	26	26	7	Anal
Kim et al.	2014	Korea	RCNS	58	58	8	Vaginal
Leung et al.	2013	China	PRCT	35	35	5^*∗*^	Anal
Ng et al.	2018	China	RCNS	35	38	8	Anal
Park et al.	2011	Korea	RCNS	34	34	7	Vaginal
Zhang et al.	2014	China	RCNS	65	132	7	Anal

Notes: NOSES, natural orifice specimen extraction surgery; CLAPS, conventional laparoscopic surgery; NOS, Newcastle-Ottawa Quality Scale; RCNS, retrospective comparative nonrandomized study; PRCT, prospective randomized controlled trial. ^*∗*^Based on the 6-item Jadad scale.

**Table 2:** Patient characteristics of the included studies.

Author	Age (year) mean ± SD		Gender (male/female)		BMI (kg/m^2^) mean ± SD		ASA (I + II/III + IV or mean ± SD)	
	NOSES	CLAPS	NOSES	CLAPS	NOSES	CLAPS	NOSES	CLAPS
Awad et al.	66.9 ± 8.9	63.6 ± 9.08	0/20	0/20	25.1 ± 6.65	31.6 ± 8.33	4/16	5/15
Hisada et al.	63.7 ± 9	66.3 ± 11	12/8	NR	NR	NR	NR	NR
Hu et al.	63.1 ± 8.3	61.5 ± 7.6	17/9	15/11	26.5 ± 4.7	26.4 ± 4.6	24/2	23/3
Kim et al.	62.8 ± 9.0	63.2 ± 10.7	0/58	0/58	23.5 ± 2.9	23.2 ± 3.3	52/6	50/8
Leung et al.	62 (51–86)^*∗*^	72 (49–84)^*∗*^	13/22	12/23	NR	NR	NR	NR
Ng et al.	65.14 ± 9.14	63.95 ± 9.19	20/15	22/16	22.64 ± 1.95	23.41 ± 1.60	2.4 ± 0.62	2.5 ± 0.47
Park et al.	61.0 ± 11.2	63.6 ± 11.6	0/34	0/34	23.9 ± 3.1	23.1 ± 2.7	30/4	28/6
Zhang et al.	56.1 ± 9.3	55.5 ± 9.5	32/33	57/75	23.7 ± 2.9	23.1 ± 3.1	60/5	116/16

Notes: NOSES, natural orifice specimen extraction surgery; CLAPS, conventional laparoscopic surgery; BMI, body mass index; ASA, American Society of Anesthesiologists; NR, not recorded; SD, standard deviation. ^*∗*^Median (range).

## Data Availability

The data used to support the findings of our article have been deposited in PubMed. All 8 articles included in our systematic review and meta-analysis can be found in PubMed; the PMID are 25469041, 24986143, 30235691, 23942527, 30705889, 24789131, 21305535, and 24566749 respectively.

## Supplementary Materials

Supplementary Materials Figure S1: subgroup analysis results of operative time: (a) based on specimen extraction route (SER), (b) based on sample size, (c) based on publication year. Figure S2: subgroup analysis results of intraoperative blood loss: (a) based on specimen extraction route (SER), (b) based on sample size, (c) based on publication year. Figure S3: subgroup analysis results of postoperative pain scores: (a) based on specimen extraction route (SER), (b) based on sample size, (c) based on publication year. Figure S4: subgroup analysis results of duration of hospital stay: (a) based on specimen extraction route (SER), (b) based on sample size, (c) based on publication year. Table S1: quality assessment based on the NOS for retrospective studies. Text S1: the search strategy of Embase.
