# Supplementary figures and images for: The Comparison of Laparoscopic Colorectal Resection with Natural Orifice Specimen Extraction versus Mini-Laparotomy Specimen Extraction for Colorectal Tumours: A Systematic Review and Meta-Analysis of Short-Term Outcomes
Source: J Oncol. 2020 Jan 31;2020:6204264. doi: 10.1155/2020/6204264 (PMC7218971; doi:10.1155/2020/6204264)

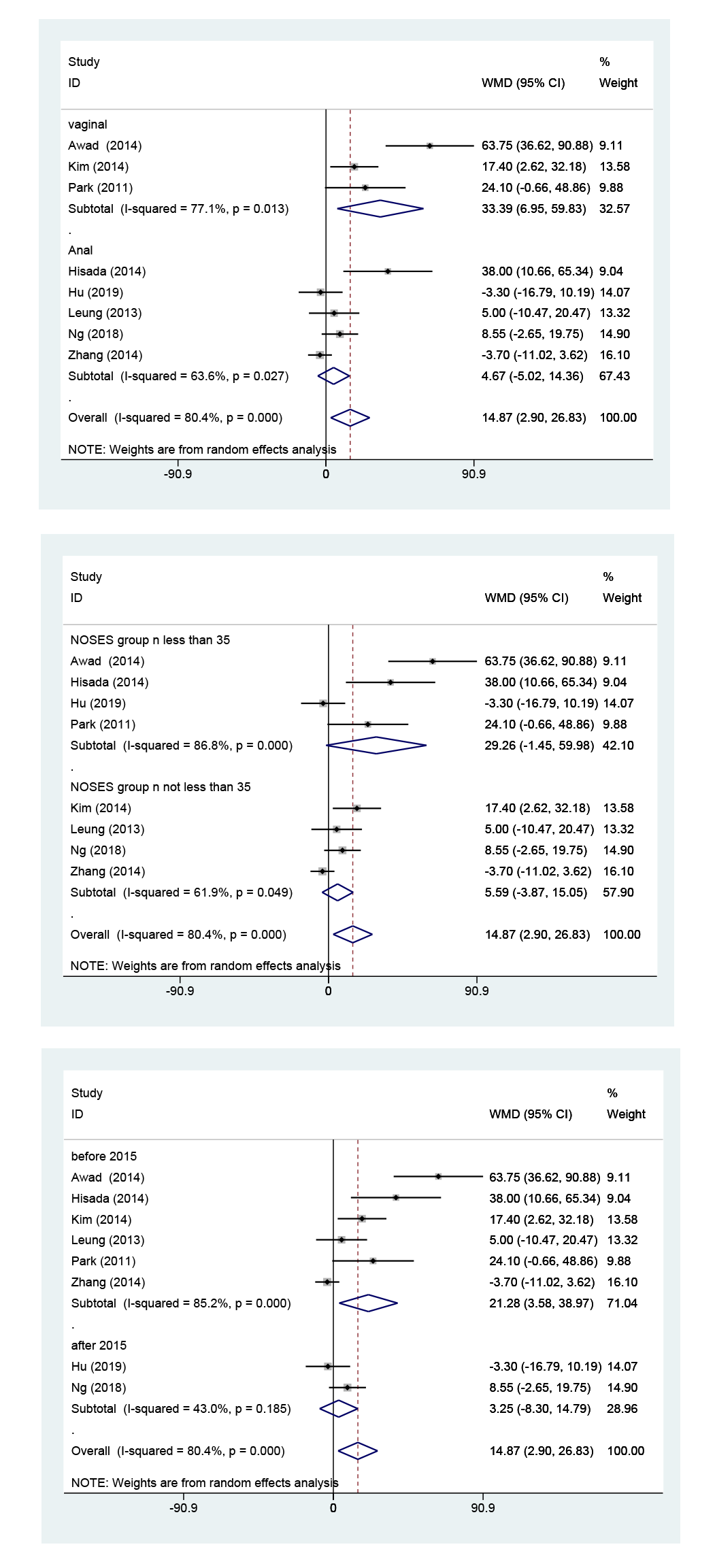

Supplement: Supplementary Materials — Figure S1: subgroup analysis results of operative time: (a) based on specimen extraction route (SER), (b) based on sample size, (c) based on publication year. Figure S2: subgroup analysis results of intraoperative blood loss: (a) based on specimen extraction route (SER), (b) based on sample size, (c) based on publication year. Figure S3: subgroup analysis results of postoperative pain scores: (a) based on specimen extraction route (SER), (b) based on sample size, (c) based on publication year. Figure S4: subgroup analysis results of duration of hospital stay: (a) based on specimen extraction route (SER), (b) based on sample size, (c) based on publication year. Table S1: quality assessment based on the NOS for retrospective studies. Text S1: the search strategy of Embase. [file 6204264.f1.zip › 6204264.f1/supplementary materials/S1_Fig.tif]

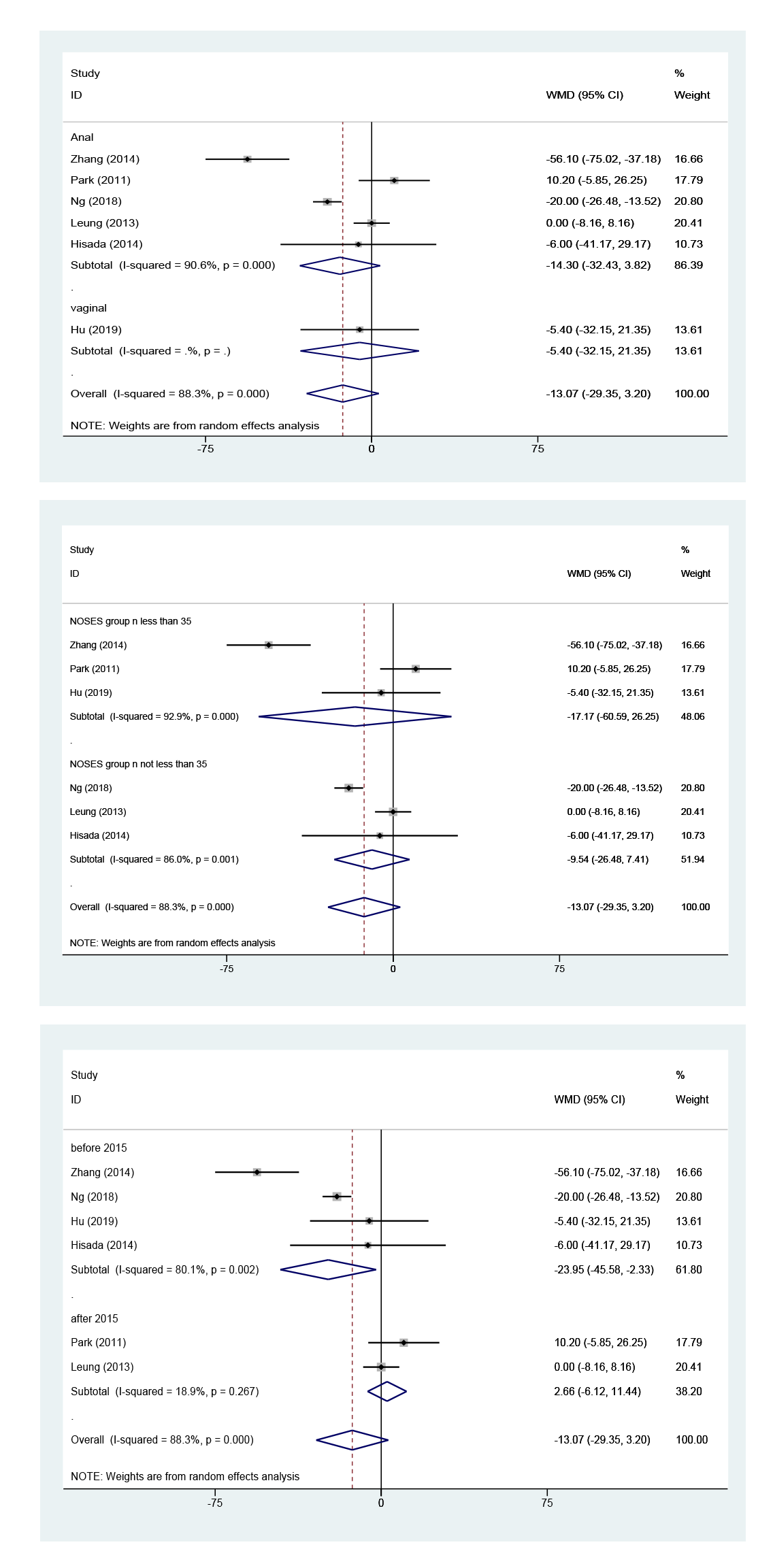

Supplement: Supplementary Materials — Figure S1: subgroup analysis results of operative time: (a) based on specimen extraction route (SER), (b) based on sample size, (c) based on publication year. Figure S2: subgroup analysis results of intraoperative blood loss: (a) based on specimen extraction route (SER), (b) based on sample size, (c) based on publication year. Figure S3: subgroup analysis results of postoperative pain scores: (a) based on specimen extraction route (SER), (b) based on sample size, (c) based on publication year. Figure S4: subgroup analysis results of duration of hospital stay: (a) based on specimen extraction route (SER), (b) based on sample size, (c) based on publication year. Table S1: quality assessment based on the NOS for retrospective studies. Text S1: the search strategy of Embase. [file 6204264.f1.zip › 6204264.f1/supplementary materials/S2_Fig.tif]

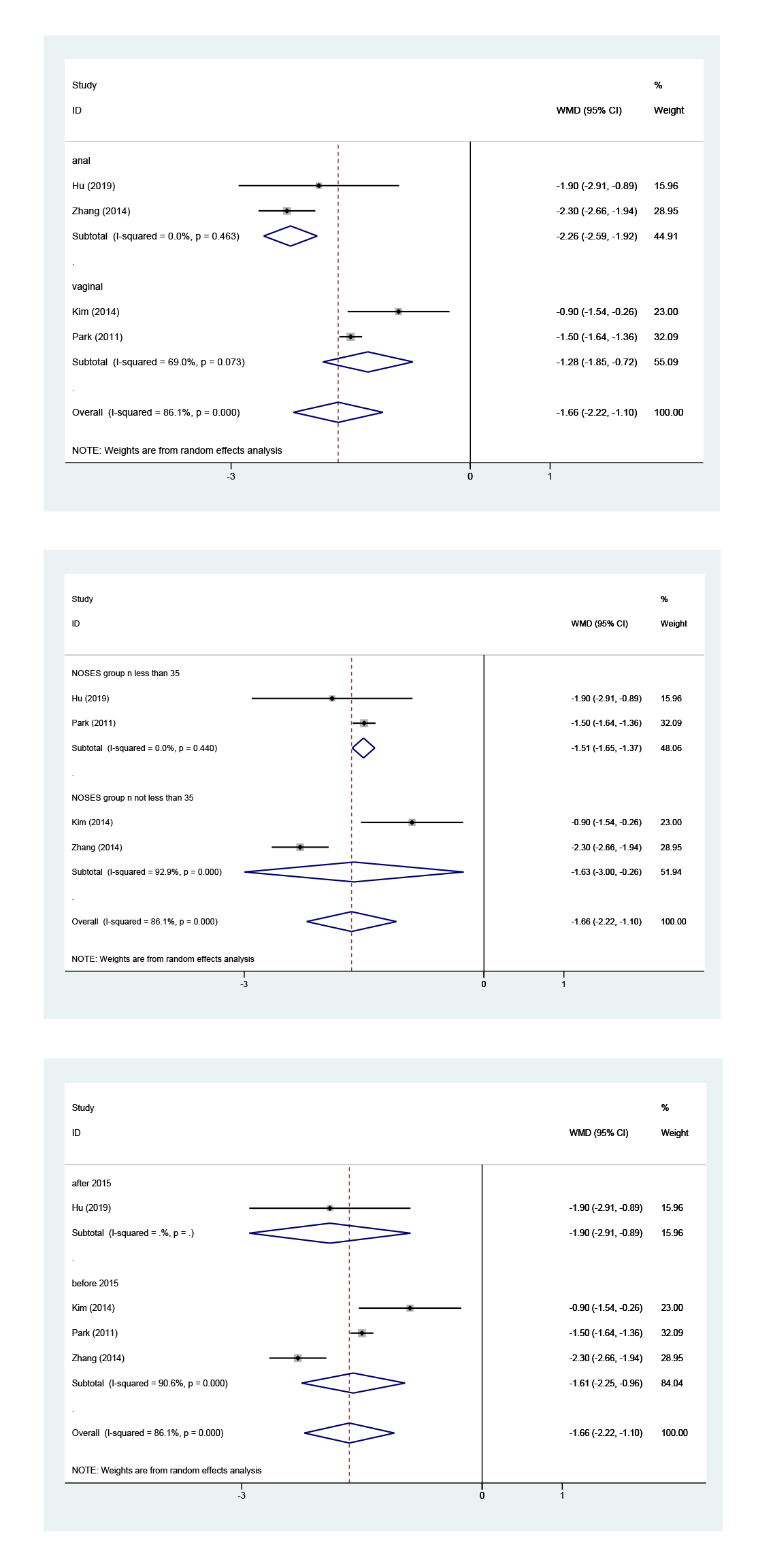

Supplement: Supplementary Materials — Figure S1: subgroup analysis results of operative time: (a) based on specimen extraction route (SER), (b) based on sample size, (c) based on publication year. Figure S2: subgroup analysis results of intraoperative blood loss: (a) based on specimen extraction route (SER), (b) based on sample size, (c) based on publication year. Figure S3: subgroup analysis results of postoperative pain scores: (a) based on specimen extraction route (SER), (b) based on sample size, (c) based on publication year. Figure S4: subgroup analysis results of duration of hospital stay: (a) based on specimen extraction route (SER), (b) based on sample size, (c) based on publication year. Table S1: quality assessment based on the NOS for retrospective studies. Text S1: the search strategy of Embase. [file 6204264.f1.zip › 6204264.f1/supplementary materials/S3_Fig.tif]

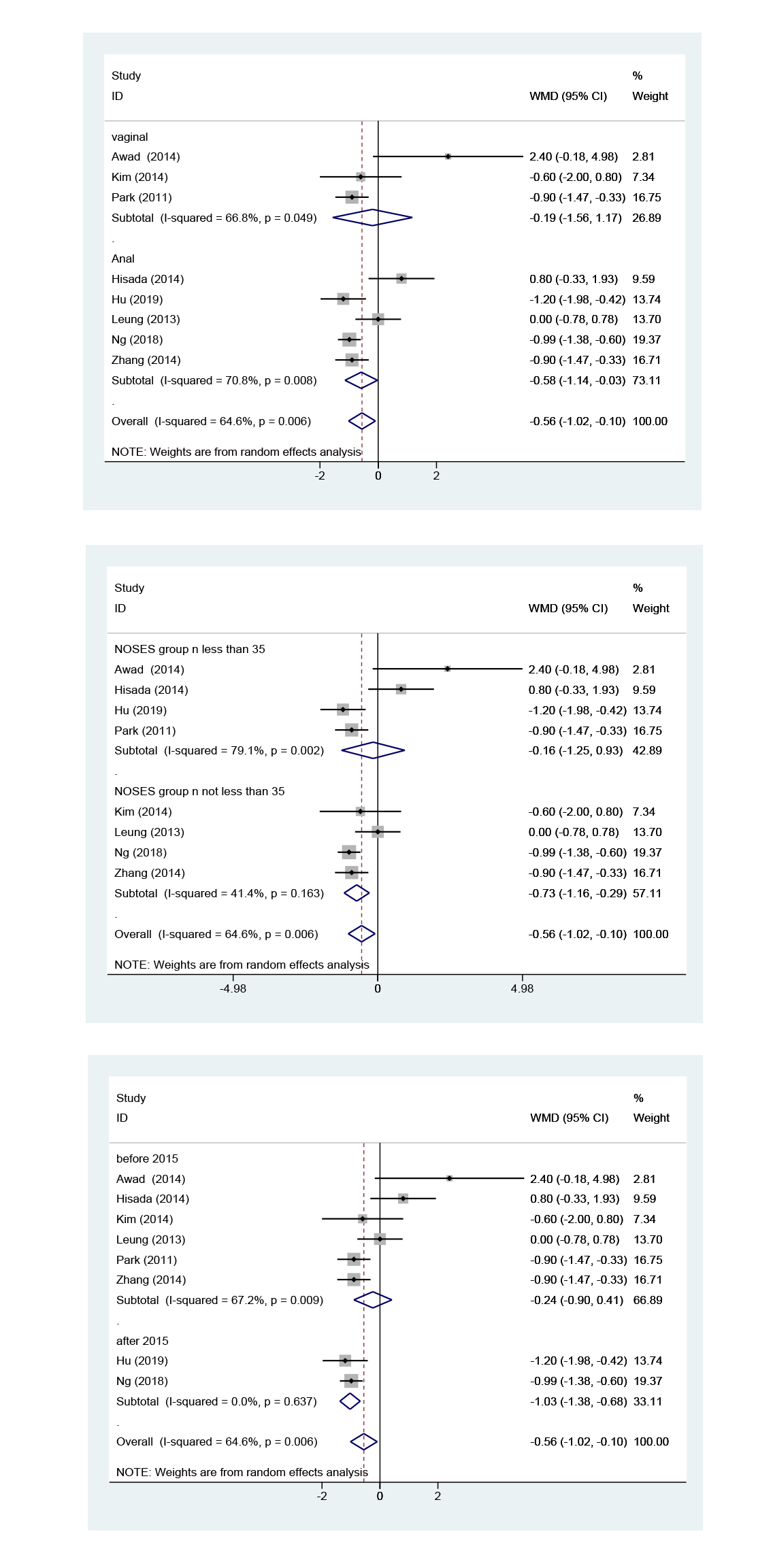

Supplement: Supplementary Materials — Figure S1: subgroup analysis results of operative time: (a) based on specimen extraction route (SER), (b) based on sample size, (c) based on publication year. Figure S2: subgroup analysis results of intraoperative blood loss: (a) based on specimen extraction route (SER), (b) based on sample size, (c) based on publication year. Figure S3: subgroup analysis results of postoperative pain scores: (a) based on specimen extraction route (SER), (b) based on sample size, (c) based on publication year. Figure S4: subgroup analysis results of duration of hospital stay: (a) based on specimen extraction route (SER), (b) based on sample size, (c) based on publication year. Table S1: quality assessment based on the NOS for retrospective studies. Text S1: the search strategy of Embase. [file 6204264.f1.zip › 6204264.f1/supplementary materials/S4_Fig.tif]
