# Supplementary material for: The Comparison of Laparoscopic Colorectal Resection with Natural Orifice Specimen Extraction versus Mini-Laparotomy Specimen Extraction for Colorectal Tumours: A Systematic Review and Meta-Analysis of Short-Term Outcomes
Source: J Oncol. 2020 Jan 31;2020:6204264. doi: 10.1155/2020/6204264 (PMC7218971; doi:10.1155/2020/6204264)
Supplement: Supplementary Materials — Figure S1: subgroup analysis results of operative time: (a) based on specimen extraction route (SER), (b) based on sample size, (c) based on publication year. Figure S2: subgroup analysis results of intraoperative blood loss: (a) based on specimen extraction route (SER), (b) based on sample size, (c) based on publication year. Figure S3: subgroup analysis results of postoperative pain scores: (a) based on specimen extraction route (SER), (b) based on sample size, (c) based on publication year. Figure S4: subgroup analysis results of duration of hospital stay: (a) based on specimen extraction route (SER), (b) based on sample size, (c) based on publication year. Table S1: quality assessment based on the NOS for retrospective studies. Text S1: the search strategy of Embase. [file 6204264.f1.zip › 6204264.f1/supplementary materials/S1_Table.doc]

| **S1 Table. Quality assessment based on the NOS for retrospective studies** | | | | | | | | | | | |
| --- | --- | --- | --- | --- | --- | --- | --- | --- | --- | --- | --- |
| Author | Selection | | | | | Comparability | | | outcome | | Score |
|  | 1 | 2 | 3 | 4 | 5 | 6 | 7 | 8 | | 9 |  |
| Awad et al. | * | * | * | * | * | * |  | * | | * | 8 |
| Hisada et al. | * | * | * | * | * |  |  | * | |  | 6 |
| Hu et al. | * | * | * | * | * | * |  | * | |  | 7 |
| Kim et al. | * | * | * | * | * | * |  | * | | * | 8 |
| Ng et al. | * | * | * | * | * | * |  | * | | * | 8 |
| Park et al. | * | * | * | * | * | * |  | * | |  | 7 |
| Zhang et al. | * | * | * | * | * | * |  | * | |  | 7 |
| Notes: NOS Newcastle-Ottawa Quality Scale, 1 representativeness of the exposed cohort, 2 Selection of the non exposed cohort, 3 ascertainment of exposure, 4 outcome was not present at start of study, 5 comparability of the most important factor, 6 comparability of second important factor, 7 assessment of outcome, 8 follow-up long enough, 8 adequacy of follow up | | | | | | | | | | | |
|  |  |  |  |  |  |  |  |  |  |  |  |
|  |  |  |  |  |  |  |  |  |  |  |  |
